# Iron nanoparticles prepared from South African acid mine drainage for the treatment of methylene blue in wastewater

**DOI:** 10.1007/s11356-024-33739-3

**Published:** 2024-05-27

**Authors:** Leo Folifac, Alechine E. Ameh, Jennifer Broadhurst, Leslie F. Petrik, Tunde V. Ojumu

**Affiliations:** 1 Department of Chemical Engineering, Cape Peninsula University of Technology, Symphony Way, Bellville 7535, PO Box 1906, Bellville 7535, Cape Town, South Africa; 2 Minerals to Metals, Department of Chemical Engineering, University of Cape Town, Woolsack Drive, Rondebosch 7701, PO Box X3, Rondebosch 7701, Cape Town, South Africa

**Keywords:** Acid mine drainage, Sodium borohydride, *Green tea extract*, Synthesis, *Iron nanoparticles*, Methylene blue, Decoloration

## Abstract

In this study, three acid mine drainage (AMD) sources were investigated as potential sources of iron for the synthesis of iron nanoparticles using green tea extract (an environmentally friendly reductant) or sodium borohydride (a chemical reductant). Electrical conductivity (EC), total dissolved solids (TDS), dissolved oxygen (DO), oxidation–reduction potential (ORP), ion chromatography (IC), and inductively coupled plasma-mass spectroscopy (ICP-MS) techniques were used to characterize the AMD, and the most suitable AMD sample was selected based on availability. Additionally, three tea extracts were characterized using ferric-reducing antioxidant power (FRAP) and 2,2-diphenyl-1-picryl-hydrazine-hydrate (DPPH), and the most suitable environmentally friendly reductant was selected based on the highest FRAP (1152 µmol FeII/g) and DPPH (71%) values. The synthesized iron nanoparticles were characterized and compared using XRD, STEM, Image J, EDS, and FTIR analytical techniques. The study shows that the novel iron nanoparticles produced using the selected green tea (57 nm) and AMD were stable under air due to the surface modification by polyphenols contained in green tea extract, whereas the nanoparticles produced using sodium borohydride (67 nm) were unstable under air and produced a toxic supernatant. Both the AMD-based iron nanoparticles can be used as Fenton-like catalysts for the decoloration of methylene blue solution. While 99% decoloration was achieved by the borohydride-synthesized nanoparticles, 81% decoloration was achieved using green tea-synthesized nanoparticles.

## Introduction

Mining activities significantly contribute to the national GDP of many countries (Sorensen 2011). As a result, mining produces large amounts of waste, which harms the environment (McCarthy 2011; McCarthy and Humphries 2013). Throughout the life of the mine, exposure of the pyrite containing overburden to oxygen occurs, resulting in the formation of acid mine drainage (AMD), a polluted effluent. AMD is formed when exposed pyritic rocks (FeS_2_) spontaneously oxidize in the presence of air and water. Additionally, when AMD is released into the environment, it can have a negative impact on the ecosystems. Noticeably, AMD pollutes rivers, kills aquatic life, and pollutes the food chain with metals (Hogsden and Harden 2012; Simate and Ndlovu 2014). Meanwhile, various reducing reagents have been used to chemically treat AMD to precipitate metals such as iron. AMD remediation and containment, possible treatments, and recovery of resources are topics of research interest (Kefeni et al. 2017; Naidu et al. 2019).

The textile industry, which uses a high proportion of colorants, produces a significant amount of toxic dye wastewater (Sivaram et al. 2018). This toxicity may contaminate soil or rivers, causing harm to aquatic life. Many countries require the treatment of dye wastewater, with an emphasis on decoloration, prior to its release into the environment. In recent years, the adsorption method for treating textile wastewater has grown in popularity. This is due to its efficacy in removing pollutants that are difficult to treat with biological methods (Pearce et al. 2003; Holkar et al. 2016). In line with this investigation, environmental studies have recently focused on using nanoparticles as an adsorbent or a Fenton-like catalyst due to their effective reactivity with contaminated textile effluent (Vadapalli et al. 2010; Humphries et al. 2017). Methylene blue is a dye that is commercially produced by using sodium dichromate (Na_2_Cr_2_O_7_) in the oxidation of N, N-dimethyl-phenylenediamine in the presence of sodium thiosulfate (Na_2_S_2_O_3_), which is followed by further oxidation with N, N-dimethylaniline. This dye is used by the textile industrys to dye cotton, wool, silk, and leather. Furthermore, the presence of methylene blue in wastewater after the dyeing process raises environmental concern. This is due to the unpleasant color it produces, its low biodegradability, and its stability in wastewater, which may cause significant environmental harm if improperly disposed of (Khodaie et al. 2013; Mashkoor and Nasar 2020; Al-Aoh et al. 2020; Muniyandi et al. 2021; Badawi et al. 2022; Teixeira et al. 2022). The performance of heterogeneous catalysts in the treatment of textile wastewater under neutral conditions decreases because of their strong pH dependence (Ouyang et al. 2019). However, recent research has shown that using a co-catalytic heterogeneous catalyst (CoFe_2_O_4_/MoS_2_) in the Fenton reaction, which is pH-independent, improves organic pollutant removal (Yan et al. 2021). Iron nanoparticles are another type of catalyst that can be applied in pollutant degradation in wastewater, and their synthesis, applications, and evaluation are well documented (Stefaniuk et al. 2016; Kumar et al. 2016; Sun et al. 2016). Their effectiveness in removing pollutants from wastewater is due to their high surface area and small particle size. However, the synthesis of iron nanoparticles is expensive since it requires the use of a chemical reductant (such as sodium borohydride), which has stringent environmental restriction policies (Kimpiab et al. 2022; Böke et al. 2023). In this line of investigation, polyphenols derived from plant extracts provide an environmentally friendly, cheap, and effective reductant for the synthesis of iron nanoparticles (Fazlzadeh et al. 2017; Abdelfatah et al. 2021). One of the most recent studies worth noting is the use of *Ricinus communis* aqueous seed extract to produce iron nanoparticles from reagent-grade iron salt that was used to treat methylene blue polluted water. Onion peel extract, extract of pomegranate, and extract of *Cleistocalyx operculate* leaf are some other plant extracts that have been studied as reductants.

While the current trend of using plant extract rather than chemical reductants (e.g., sodium borohydride) for the synthesis of iron nanoparticles from reagent-grade iron salt addresses a portion of the growing environmental concerns, investigating various methods of recovering Fe from AMD in the form of iron nanoparticles would not only reduce the cost of synthesizing iron nanoparticles and treating AMD, but it would also promote sustainable development thinking in the design of such processes. The goal of this research is to synthesize high-quality iron nanoparticles from AMD using an environmentally friendly reductant such as green tea extract, which has higher FRAP and DPPH values than other tea extracts described in this study. The view is to compare the tea-synthesized iron nanoparticles from AMD with those synthesized from AMD using sodium borohydride in terms of their characteristics and performance with respect to catalytic activities for the decoloration of methylene blue in wastewater.

## Materials and methods

### Chemicals and feedstock collection

Nitric acid (HNO_3_; 99.9%), sodium borohydride (NaBH_4_; 99.8%), and ethanol (C_2_H_5_OH; 50%) were purchased from Kimix, South Africa; meanwhile, green tea (*Camellia sinensis*), rooibos tea (*Aspalathus linearis*), and green rooibos tea (*A. linearis*) were purchased from a supermarket (Shoprite). The as-received field AMD samples (feedstock solutions) were obtained from the Navigation coal mine sites in the Mpumalanga Province. The feedstock solutions were filtered using a 0.45-µm membrane filter paper and stored in an airtight container to prevent the ingress of air that could change the composition of the solution.

### Reductant preparation

Green reductants (such as green tea, rooibos tea, or green rooibos tea extracts) and sodium borohydride were prepared for the reduction of iron from AMD. In each case, 60 g of dry tea leaves were powder-ground and mixed with 300 mL of deionized water and 100 mL of a 25% ethanol solution. The separate tea mixtures were heated at 70 °C for 60 min while being stirred with a magnetic bar. After the time had elapsed, the tea mixtures were removed, cooled, and filtered using a 0.45-µm membrane filter paper. Following that, the ethanolic content of the extracted solutions was evaporated using a rotor vapor set at 65 °C. The water content in the separate tea crude was removed using a freeze dryer to obtain 15 g of the tea powder. In each case, the obtained amorphous powder was subjected to characterization and antioxidant tests prior to the selection of the tea extract with the highest antioxidant capacity. 0.1 to 0.7 g of the selected extract (green tea) was measured separately and mixed with 50 mL of deionized water for 1 min. In each case, the mixture was centrifuged at 5000 rpm for 5 min (to sediment any tiny, suspended particles) prior to filtration using a 0.45 µm membrane filter paper to obtain a green tea extract solution. Similarly, 0.1 to 0.7 M solutions of sodium borohydride were prepared from powdered sodium borohydride (NaBH_4_).

### Reductant Antioxidant Test

The prepared tea reductants were physically and chemically tested, and their antioxidant potential was determined. The 2,2-diphenylpicrylhydrazyl (DPPH) antioxidant test was conducted using the method developed by Badmus et al. (2018). 200 mg/L of green tea, green rooibos tea, or rooibos tea extract solution was prepared and serially diluted to 100, 50, 25, 12.5, 6.25, and 3.12 mg/L. Similarly, 200 mg/L stock solution of ascorbic acid was prepared and serially diluted to 100, 50, 25, 12.5, 6.25, or 3.12 mg/L. Blank test samples were prepared by mixing 100 µL of the serially diluted tea extracts with 50 µL of absolute methanol. In a similar manner, a negative control solution was prepared by mixing 100 µL of 150 mg/L DPPH with 50 µL of absolute methanol. Following that, 50 µL of the mixture was measured and transferred to 100 µL of serially diluted tea extracts, ascorbic acid, blanks, and negative controls on a microplate that was kept in the QQN for 30 min. The microplate absorbance was measured, and the experiments were performed in triplicate. Equation 1 was used to convert the values to percentage inhibition.

1 $$\% inhibition=\frac{Abs.blank \times Abs.Sample}{Abs.blank}\times 100$$

The ferric ion reducing antioxidant power (FRAP) of the tea extracts was determined using the method that was developed by Benzie and Strain (1996) with minor modifications. A buffer acetate solution of 300 mM (Millimolar), pH 3.6, and a solution of 10 Mm TPTZ (2,4,6-tripyridyl-s-triazine) were mixed in the presence of 0.1 M HCl and FeCl_3_⋅6H_2_O (20 mM) in a ratio of 10:1:1 to obtain an active FRAP. Thereafter, in each case, 2 mg/L of ascorbic acid (standard) and plant extract were prepared in a careful manner and immediately subjected to 5 min of agitation using a vortex mixer (Dragon LAB MX-S). The agitated mixture was then centrifuged using an Eppendorf centrifuge 5810R for 5 min at 1000 rpm to permit the formation of clear solutions of the test samples. Subsequently, 100 mL of the tea extract solution was separately mixed with 300 mL of the prepared active FRAP reagent. The calibration curve for the known Fe^2+^ concentration was created by using methanol solutions of FeSO_4_⋅7H_2_O ranging from 100 to 2000 μM. The parameter equivalent concentration is defined as the concentration of antioxidant having a Ferric-TPTZ reducing ability equivalent to that of 1 mM FeSO_4_⋅7H_2_O.

### Synthesis of *iron* nanoparticles from AMD using green tea extract (GTE)

100 mL of AMD solution was measured and purged with nitrogen gas for about 15 min to remove dissolved oxygen. Thereafter, the purged AMD solution was subjected to magnetic stirring at 250 rpm. In each case, a separately prepared 50 mL solution containing 0.1 to 0.7 g of powdered green tea extract was added in a dropwise manner to AMD to precipitate iron nanoparticles, and the mixture was stirred for 8 h at 40 °C (with the view to selecting the extract that gives the highest recovery). The color of the solution changed from yellow to black, indicating the presence of iron nanoparticles. The mixture was then filtered using 0.22-µm filter paper to separate the iron nanoparticles from the supernatant, followed by washing and then drying in an inert environment. The obtained iron-depleted supernatant solution was characterized using IC and ICP-MS, whereas the synthesized iron nanoparticles were characterized using XRD, SEM/EDS, STEM, Image J, and FTIR. The effects of temperature and contact time on nano-iron recovery from AMD were individually investigated to obtain the optimum temperature and time.

### Synthesis of *iron* nanoparticles from AMD using sodium borohydride

100 mL of AMD solution was measured and purged with nitrogen gas for about 15 min to remove dissolved oxygen. Thereafter, the purged AMD solution was subjected to magnetic stirring at 250 rpm. In each case, a separately prepared 50 mL solution of 0.1 to 0.7 M sodium borohydride dosage was added to AMD in a dropwise manner to precipitate iron nanoparticles and then mixed for 80 min at 25 °C (with the view to selecting the concentration that gives the highest recovery). The color of the solution changed to black with the evolution of a colorless gas. The mixture was then filtered using 0.22-µm filter paper to separate the iron nanoparticles from the supernatant, followed by washing and then drying in an inert environment. The generated iron-depleted supernatant solution was characterized using IC and ICP-MS, whereas the synthesized iron nanoparticles were characterized using XRD, SEM/EDS, STEM, Image J, and FTIR. Optimal contact time and temperature were used during synthesis (Dhanemozhi et al. 2017). Equations 2 and 3 summarize the synthesis of iron nanoparticles from AMD using sodium borohydride.

2 $$2F{e}^{2}+ + {H}_{2}{O}_{2(aq)} + {2{H}^{+}}_{(aq)} \to 2F{e}^{3}+ + 2{H}_{2}{O}_{(\text{l})}$$

3 $$4F{e}^{3+} + {3B{H}_{4}}^{-} + 9{H}_{2}O \to 4F{e}^{0} + {3{H}_{2}B{O}_{3}}^{-} + 12{H}^{+} + 6{H}_{2}$$

### Characterization of *iron* nanoparticles from AMD

The mineralogy of the synthesized iron nanoparticles was investigated using X-ray diffraction (Phillip X-pert pro-MPD X-ray diffractometer). The morphology and elemental composition of the nanoparticles were obtained using scanning electron microscopy (SEM, Hitachi 650). The surface morphology and particle size of the nanoparticles were investigated using scanning transmission electron microscopy (STEM; TECNAI G2 F20 X Twin Mat FEG) and an Image J particle size analyzer. The molecular structure of the iron nanoparticles and green tea extract was determined using the FTIR (Perkin Elmer 1600 analyzer). The chemical composition of the AMD samples, tea extracts, and AMD supernatants was analyzed using ion chromatography (IC, LICS-A20) and inductively coupled plasma-mass spectroscopy (ICP-MS 7900 by Agilent).

### Treatment of methylene blue solution using *iron* nanoparticles synthesized from AMD

The AMD-based green tea synthesized iron nanoparticles synthesized at optimum concentration, time, and temperature (GTNI 0.5) or the AMD-based sodium borohydride synthesized iron nanoparticles at optimum concentration and time (SBNI 0.6) were used to decolorize simulated methylene effluent. In each case, 100 mL (10 ppm) of simulated methylene blue solution was mixed with 0.001 M HCl and 50% hydrogen peroxide to prepare solutions with pH values from 1 to 5. Each solution was tested with 5–15 mg of GTNI 0.5 or SBNI 0.6 for 30–180 min in a shaker. The percentage decoloration of methylene blue was analyzed using ultraviolet and visible spectrometry (UV–Vis) and was compared with the control reaction (prepared with 100 mL (10 ppm) of a simulated MB solution, 0.001 M HCl, 50% hydrogen peroxide, and green tea extract). The decoloration of methylene blue in the solution was calculated using Eq. 4:

4 $$\%Removal\;efficiency=\frac{C_o-C_f}{C_o}\times100$$

where *C*_0_ (mg/L) is the initial concentration of methylene blue and *C*_*f*_ (mg/L) is the concentration of methylene blue after a certain time.

## Results and discussion

### Physical and chemical characterization of AMD and tea extracts

Table 1 showed both physical and chemical characteristics of AMD and tea extract.

**Table 1:** Physical and chemical characteristics of acid mine drainage (AMD) feedstock and tea extracts

Elements	Unit	AMD			Tea		
		Kopseer	Toeseep	Penstock	Green tea	Rooibos tea	Green rooibos tea
**Anions**							
SO_4_^2−^	mg/L	13,200 ± 72.3	11,500 ± 29.0	9910 ± 68.1	-	-	-
NO_3_^−^	mg/L	3.89 ± 1.0	2.13 ± 0.2	2.20 ± 1.2	-	-	-
Cl^−^	mg/L	15.3 ± 0.2	2.20 ± 0.20	10.1 ± 1.2	-	-	-
**Metals**							
Fe	mg/L	4420 ± 14.3	1930 ± 10.8	4002 ± 22.1	0.9	0.12	0.89
Ca	mg/L	527 ± 22.4	508 ± 19.3	503 ± 18.9	4.32	8.21	7.10
Mg	mg/L	575 ± 8.21	414 ± 4.46	465 ± 15.2	-	1.1	0.21
Al	mg/L	460 ± 18.2	246 ± 3.58	367 ± 21.2	12.0	11.31	14.29
Mn	mg/L	126 ± 1.21	48.3 ± 0.95	85.4 ± 3.8	-	-	-
K	mg/L	BDL	BDL	BDL	281	311	297
Na	mg/L	33.5 ± 1.21	64.2 ± 0.21	33.1 ± 0.92	1.44	3.21	2.21
Si	mg/L	33.8 ± 2.55	12.0 ± 0.08	28.1 ± 1.02	0.97	4.39	2.41
Zn	mg/L	13.1 ± 0.07	6.10 ± 0.05	9.8 ± 0.01	0.29	0.41	0.43
As	mg/L	0.16 ± 0.07	0.21 ± 0.01	0.32 ± 0.02	-	-	-
B	mg/L	0.11 ± 0.02	0.17 ± 0.04	0.14 ± 0.03	-	-	-
Be	mg/L	0.15 ± 0.08	0.13 ± 0.05	0.14 ± 0.04	-	-	-
Cd	mg/L	0.001 ± 0.01	0.001 ± 0.03	0.001 ± 0.01	-	-	-
Co	mg/L	1.84 ± 0.08	0.52 ± 0.02	1.11 ± 0.05	0.008	0.57	0.87
Cr	mg/L	0.42 ± 0.22	0.51 ± 0.09	0.34 ± 0.07	-	-	-
Cu	mg/L	0.11 ± 0.04	0.18 ± 0.01	0.11 ± 0.02	0.12	0.02	0.19
Hg	mg/L	0.01 ± 0.01	0.01 ± 0.04	0.01 ± 0.02	-	-	-
Mo	mg/L	0.08 ± 0.01	0.09 ± 0.01	0.08 ± 0.06	-	-	-
Ni	mg/L	1.87 ± 0.02	0.97 ± 0.03	1.12 ± 0.07	0.15	0.28	0.19
P	mg/L	0.35 ± 0.08	0.20 ± 0.01	0.001 ± 0.03	62.0	73.1	71.4
Pb	mg/L	0.02 ± 0.05	0.04 ± 0.03	0.16 ± 0.01	-	-	-
Sb	mg/L	0.02 ± 0.02	0.02 ± 0.02	0.02 ± 0.03	-	-	-
Sr	mg/L	0.36 ± 0.01	0.38 ± 0.09	0.84 ± 0.05	0.02	0.01	0.31
Th	mg/L	0.02 ± 0.01	0.001 ± 0.03	0.01 ± 0.01	-	-	-
Ti	mg/L	0.10 ± 0.04	0.10 ± 0.07	0.1 ± 0.05	-	-	-
V	mg/L	0.11 ± 0.04	0.12 ± 0.01	0.22 ± 0.09	-	-	-
Y	mg/L	2.34 ± 0.04	1.07 ± 0.08	1.91 ± 0.06	-	-	-
Zr	mg/L	0.01 ± 0.02	BDL	0.01 ± 0.03	-	-	-
**pH**		2.08 ± 0.02	2.20 ± 0.03	2.14 ± 0.02	5.33	5.22	5.10
**EC**	mS/cm	47 ± 0.09	15 ± 0.04	23 ± 0.02	800	700	750
**TDS**	mg/L	78 ± 0.08	29 ± 0.06	55 ± 0.05	-	-	-
**DO**	mg/L	1.2 ± 0.04	1.8 ± 0.03	1.5 ± 0.01	-	-	-
**ORP**	**mV**	458 ± 0.04	425 ± 0.01	448 ± 0.03	179	205	210

From Table 1, the results showed that the various AMD samples have low pH values, with the Kopseer AMD having the highest acidity. The acidity of the Kopseer AMD can be linked to its lower pH, which occurred as a result of the dissolution of pyrite ores into solution, resulting in more free ions in the AMD (Akcil and Koldas 2006). The dissolved oxygen (DO) of the Toeseep AMD was found to be higher than that of the Penstock and Kopseer AMD, and their corresponding water quality is arranged in descending order: Toeseep > Penstock > Kopseer. Literature also suggests that a decrease in DO can be attributed to the oxidation of Fe^2+^ to Fe^3+^, which produces AMD rich in hydrogen ions and, as a result, increases the acidity of the solution with the release of metal ions (Akcil and Koldas 2006). The redox potential values, ORP (mV, Ag/AgCl), of all the samples are indicative that the iron is predominantly in the form of ferric ions.

The AMD samples contained major, minor, and trace elements (Kalombe et al. 2020). Fe was found to be the dominant metal, and sulfate was the dominant anion in the AMD samples (Alegbe et al. 2019). In this study, Penstock AMD was the selected sample used for nano-iron synthesis. This was due to its availability and high Fe concentration.

Similar acidic pH values were noticed for the tea extracts in Table 1. This aligns with similar acidic pH values from previous research for rooibos tea, rosehip tea, black Assam tea, and green mee tea (Jaganyi and Wheeler 2003). Table 1 also depicted that green tea extract had the highest EC value and the lowest redox potential when compared to its counterpart. This meant that the green tea solution had more free ions, which made it a stronger reducing agent (Dhanemozhi et al. 2017; Widatalla et al. 2022). This could also mean that green tea extract contains more redox species in the solution (Vishnoi et al. 2018). The most dominant metals in the solution of the tea extracts were observed to be K, P, Al, and Ca.

### Antioxidant activity of tea extracts

Following the extraction of tea extracts from dried ground tea leaves, their antioxidant properties were obtained using the FRAP and DPPH assay methods. This technique was used to determine if the antioxidant capacity of the tea extracts could reduce iron in the solution. The FRAP test uses the polyphenolic compound in the tea extract to reduce ferric to ferrous ions that, in turn, form a colored complex termed ferrous-tripyridyl triazine complex. The FRAP data in Table 2 were then calculated by comparing the absorbance change at 593 nm in the test mixture with those containing ferrous ions per dry weight of the tested sample. In a similar manner, the scavenging radical activity of the tea extracts was determined by reacting a hydrogen-donating antioxidant compound (tea extracts) with DPPH. The DPPH values in Table 3 were then calculated by monitoring the change in absorbance at 517 nm as the color changed from violet to yellow.

**Table 2:** Ferric-reducing antioxidant power (FRAP) values of ethanolic tea extracts used in this study

Plant and chemical reductants	FRAP per dry weight of sample (µmol FeII/g)	Coefficient of variation (%) for triplicate measurement
Green tea extract	1152	1.37
Green rooibos tea extract	801	1.05
Rooibos tea extract	733	1.24
Sodium borohydride	6004	2.67

**Table 3:** 2,2-diphenyl-1-picryl-hydrazyl-hydrate (DPPH) values of ethanolic tea extracts used in this study.

% inhibition				
Antioxidant (mg/L)	Ascorbic acid	Green T	Green rooibos T	Rooibos T
3.12	25.3 ± 0.23	2.71 ± 0.91	1.12 ± 1.21	1.57 ± 0.02
6.25	41.83 ± 1.48	3.16 ± 0.06	2.11 ± 1.42	2.86 ± 1.11
12.5	66.84 ± 2,67	17.38 ± 0.21	11.37 ± 0.98	1.31 ± 0.71
25	96.52 ± 0.98	24.49 ± 0.9	19.41 ± 1.42	8.94 ± 1.71
50	96.97 ± 1.21	40.92 ± 1.1	40.11 ± 2.41	17.81 ± 2.11
100	97.01 ± 0.72	71.65 ± 1.4	65.92 ± 3.1	34.40 ± 1.98

*T* tea

Table 2 revealed that the FRAP values of green tea extract, green rooibos tea extract, rooibos tea extract, and sodium borohydride were 1152, 801, 733, and 6004 mol FeII/g, respectively, with sodium borohydride having the highest FRAP value for the reduction of Fe, followed by green tea extract. However, the various tea extracts examined in this study could reduce iron. The obtained FRAP results are in accordance with the findings by Jacqueline (2013) that the tea extracts contain sufficiently polyphenolic compounds with the ability to act as a reducing agent by donating electrons to reduce iron.

The scavenging antioxidant activities of the plant reductants were measured against stable free radicals in DPPH. The various tea extracts showed a high percentage of inhibition at 100 mg/L on DPPH (see Table 3). However, ascorbic acid as a reference standard (reductant) proves to have a higher percentage of inhibition that is greater than 97% at 100 mg/L for DPPH. Even though ascorbic acid has a higher percentage inhibition, the percentage of inhibition values for the tea extracts (green tea and green rooibos tea) were significant, although somewhat lower compared to that of ascorbic acid as a reference reductant. These results are in line with findings in the literature (Badmus et al. 2018). Consequently, green tea, green rooibos tea, and rooibos tea extracts can inhibit oxidative processes through significant reactions in a biological matrix. Among the characterized reductants, green tea extract was selected as the choice of reductant for the reduction of Fe from AMD. This is because the FRAP and DPPH values of green tea extract were higher than those of rooibos and green rooibos tea extracts.

### Parameters affecting the removal of Fe from AMD

GTNI iron nanoparticles were synthesized using AMD and green tea extract. In each case, 50 mL of a green tea extract solution containing powdered green tea extract (0.1, 0.2, 0.3, 0.4, 0.5, 0.6, or 0.7 g) was mixed with 100 mL of AMD at optimal time (8 h) and temperature (40 °C) to precipitate iron nanoparticles. The goal is to select the extract with the highest recovery. SBNI iron nanoparticles were synthesized using AMD and sodium borohydride. In each case, 50 mL of a sodium borohydride solution (0.1, 0.2, 0.3, 0.4, 0.5, 0.6, or 0.7 M) was mixed with 100 mL of AMD at room temperature and optimum time (1 h and 20 min). This precipitated iron nanoparticles. The goal was to select the concentration with the highest recovery. The iron nanoparticles with the maximum recovery employing green tea extract or sodium borohydride were assigned the codes GTNI 0.5 or SBNI 0.6, respectively.

**Fig. 1:**
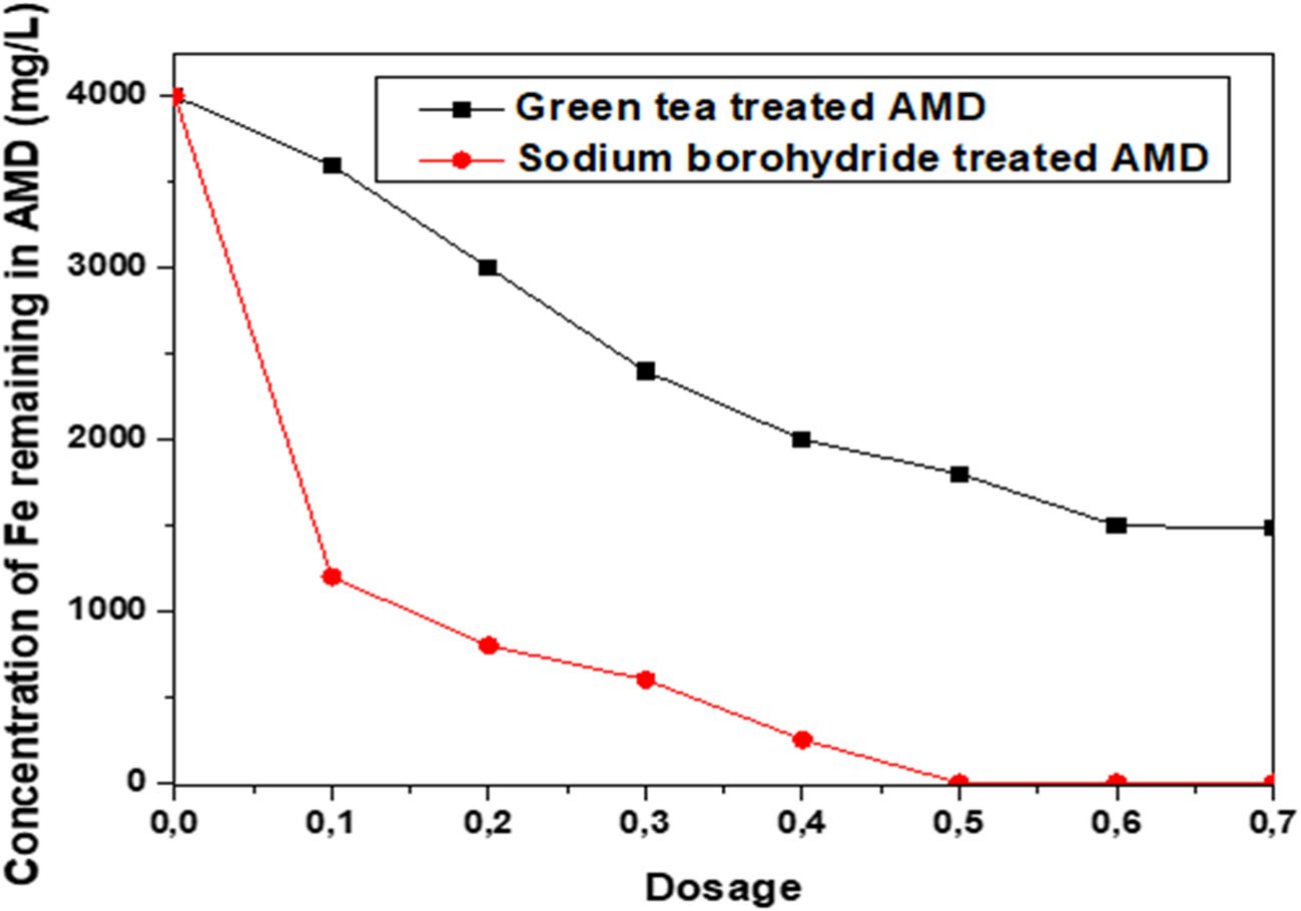
The effect of dosage of reductant on the removal of Fe as iron nanoparticles from AMD

Figure 1 depicts the trend of Fe removal from AMD using various parameters. Varying the dosage of green tea extract at optimum temperature (40 °C) and time (8 h) decreased the concentration of Fe in AMD from 4002 to 1500 mg/L, whereas varying the concentration of sodium borohydride at optimum temperature (25 °C) and time (1 h 20 min) decreased the concentration of Fe in AMD from 4002 to 0.06 mg/L.

The reported method used to remove Fe as nanoiron from AMD using sodium borohydride as a reductant was obtained from Alegbe et al. (2019), with a slight modification. In summary, when sodium borohydride was used to reduce Fe from AMD, almost 100% of Fe was removed from the solution, as opposed to 62% Fe removal when green tea extract was used.

### Characterization of *iron* nanoparticles synthesized from AMD

**Fig. 2:**
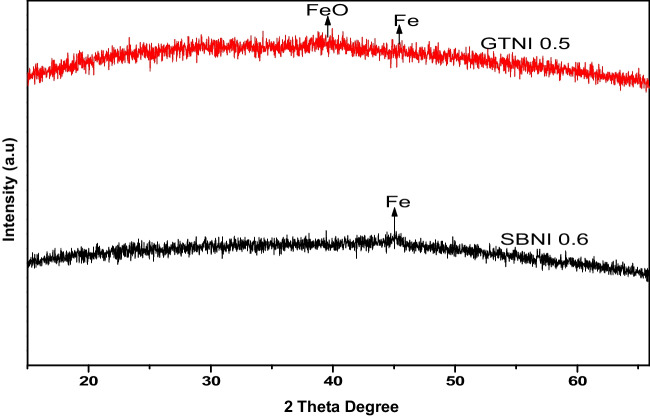
X-ray diffraction spectra of **a** green tea synthesized iron nanoparticles (GTNI 0.5), and **b** sodium borohydride synthesized iron nanoparticles (SBNI 0.6). Iron (II) oxide (FeO) or zerovalent iron (Fe) nanoparticles

Figure 2 displays the XRD of the mineralogy of iron nanoparticles synthesized from AMD using green tea extract or sodium borohydride.

The figure showed that iron (II) oxide (FeO) nanoparticles were the major mineral phase produced when green tea extract was used as a reductant. This could be attributed to the comparable low FRAP strength of green tea extract (*C. sinensis*). The broad fingerprint peaks of iron (II) oxide (FeO) or zerovalent iron (Fe) nanoparticles that were noticed at 38 or 45° 2θ were corroborated by past studies (Sun et al. 2006; Lu et al. 2007; R. Yuvakkumar et al. 2011; Ravikumar et al. 2016; Eljamal et al. 2018). In the case of using sodium borohydride as a reductant, zerovalent iron nanoparticles were mostly produced from the AMD solution, as indicated by the broad fingerprint peak at 45° 2θ. This result aligns with the results from previous studies (Ravikumar et al. 2016; Eljamal et al. 2018; Alegbe et al. 2019). The broad low-intensity peaks on both spectra are typical of the presence of nano-sized particles.

**Fig. 3:**
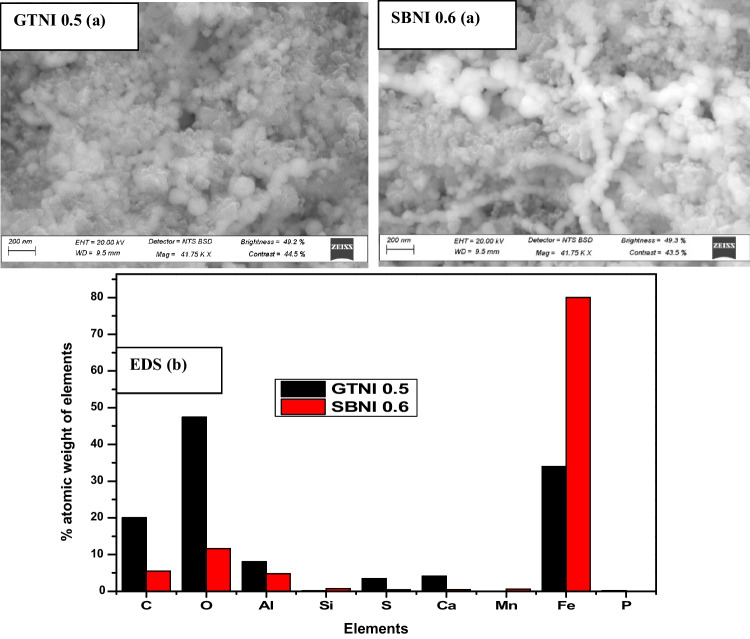
**a** Surface morphology and **b** elemental composition results of synthesized iron nanoparticles (GTNI 0.5 or SBNI 0.6)

Figure 3 presents the morphology and elemental composition of GTNI 0.5 and SBNI 0.6 nano-iron particles extracted from AMD.

The iron nanoparticles (GTNI 0.5) synthesized from AMD using green tea extract as a reductant were not clear enough to analyze morphologically, and therefore a further investigation using STEM was required, whereas the sodium borohydride synthesized iron nanoparticles from AMD were in the nano-range having a spherical beadlike structure. The SBNI 0.6 morphology aligns with the morphology obtained from the synthesis of iron nanoparticles using sodium borohydride and reagent-grade iron salt from a previous study (Badmus et al. 2018). The energy-dispersive X-ray spectroscopy (EDS) results revealed some of the elements that co-precipitated with the AMD-based iron nanoparticles (Alegbe et al. 2019). These elements either came from AMD or, to an extent, from green tea extract or sodium borohydride reductant that was used to produce GTNI 0.5 or SBNI 0.6, respectively. The high carbon content observed in GTNI 0.5 indicates the presence of polyphenols in green tea. The high Al content observed on GTNI 0.5 came from green tea extract, as supported by its ICP results. The low oxygen content in SBNI 0.6 and the high oxygen content in GTNI 0.5 corroborate the fact that SBNI 0.6 was mostly made up of nZVI particles, whereas GTNI 0.5 was mostly made of FeO nanoparticles as depicted on the XRD spectra. It is also noteworthy that the EDS analytical technique is not an accurate method for quantitatively measuring the elemental composition of a substance.

**Fig. 4:**
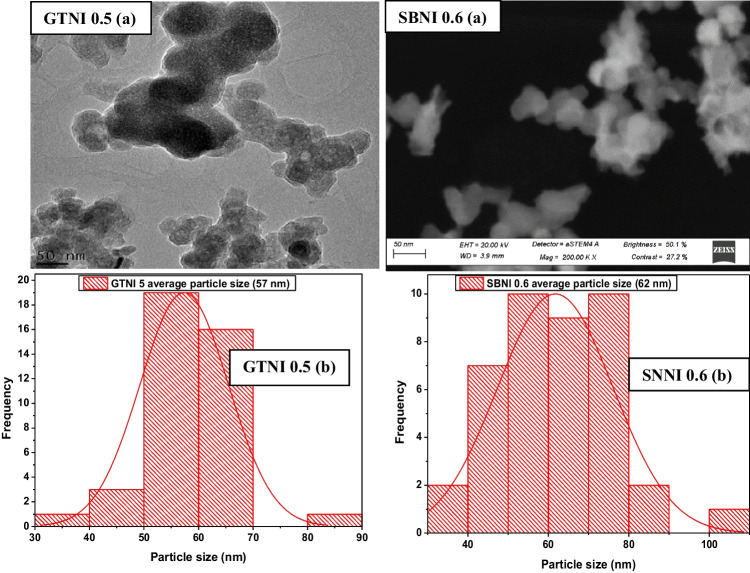
**a** STEM micrographs and **b** particle size distribution results of synthesized iron nanoparticles

Figure 4 presents the surface morphology and the particle size distribution of the synthesized iron nanoparticles (GTNI 0.5 and SBN I 0.6) from AMD.

The green synthesized iron nanoparticles (GTNI 0.5) from AMD had a spherical morphology with an average particle size of 57 nm. Huang et al. (2014) reported the production of iron nanoparticles from oolong tea extract and reagent-grade Fe salt with an average particle size of 40 to 55 nm, which is well within the range of iron nanoparticles produced in this study. SBNI 0.6 iron nanoparticles were spherical, with an average particle size of 62 nm. SBNI 0.6 iron nanoparticle morphology is consistent with the literature (Azzam et al. 2016).

**Fig. 5:**
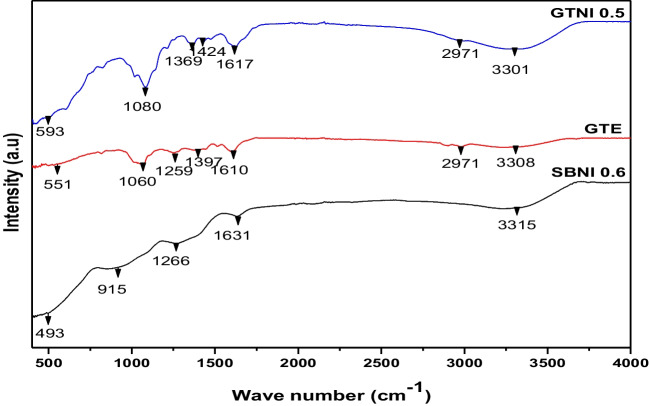
FTIR results of green tea extract (GTE), green tea synthesized nano-iron (GTNI 0.5), and sodium borohydride synthesized nano-iron (SBNI 0.6)

The structural analysis of the synthesized iron nanoparticles from AMD with assigned code names GTNI 0.5 and SBNI 0.6 is presented in Fig. 5.

The vibrations of polyphenolic compounds were observed on the FTIR spectra of green tea extract (GTE) and green tea synthesized AMD-based iron nanoparticles (GTNI 0.5). The OH stretching vibration was noticed at 3301 and 3308 cm^−1^ on GTE and GTNI 0.5, respectively. The broad OH spectrum indicates the presence of benzylic OH contributed by the plant extract’s polyphenolic content (Ashokkumar and Ramaswamy 2014; Xu et al. 2014). The peak at 2971 cm^−1^ represents C–H vibrations, which were absent on the SBNI 0.6 spectra. The less prominent peak on the spectra of GTE and GTNI 0.5 indicates the vibrations of aromatics and moisture from the air. The peaks at 1617 and 1610 cm^−1^ on the spectra of GTE and GTNI 0.5 could be assigned to aromatic skeletal vibrations. It also suggests the presence of aromatic compounds and the adherence of polyphenolic compounds to the reactive surfaces of plant-stabilized iron nanoparticles. In this line of investigation, the asymmetric, symmetric, and C = O stretching peaks were noticed at 1300–1000 cm^−1^ on GTNI 0.5 and GTE (Badmus et al. 2018). The carbonyl sugar base of the tea extract acts as a surface modifier of the synthesized iron nanoparticles that prevents agglomeration and, in the right proportion, enhances the catalytic activity of the nano-iron. SBNI 0.6 also showed vibrational bands between 500 and 3325 cm^−1^. Sundrarajan and Ramalakshmi (2012) found that the nano-iron particles synthesized from ionic liquid have bands at 360 and 570 cm^−1^ that correspond to the stretching and bending vibrations of Fe–O bonds, which are typical of magnetite’s crystalline lattice. SBNI 0.6 revealed similar Fe–O vibrational bands between 493 and 915 cm^−1^. The vibrational bands at 1650 and 3325 cm^−1^ on the SBNI 0.6 spectra are assigned to O–H bending and stretching due to Lepidrocite (ɤ-FeOOH) formation on the surface of the nano zerovalent iron (nZVI). The vibrational band at 1266 represents C–O stretching. In summary, the main structural difference between GTNI 0.5 and SBNI 0.6 is the absence of polyphenolic vibrations on the SBNI 0.6 FTIR spectra.

### Simultaneous remediation of AMD using green tea extract or sodium borohydride

AMD was used as a feedstock solution for the synthesis of iron nanoparticles using green tea extract (GTNI 0.5) or sodium borohydride (SBNI 0.6). This procedure simultaneously produced a product that is valuable and that removed various contaminants from AMD. The elemental composition, or percentage removal, is presented in Table 4.

**Table 4:** Elemental composition of AMD supernatant after the extraction of Fe from AMD using an optimized dosage of green tea extract or sodium borohydride as reductants

Parameter	Units	Raw AMD	Green tea-treated AMD	NaHB_4_-treated AMD
PH		2.14	1.81	8.58
Conductivity (EC)	mS/cm	2900		
Redox potential	mV	448	177	133
**Major elements**				
Fe	mg/L	4002 ± 22.1	1500	0.06
Ca	mg/L	503 ± 18.9	395	313
Mg	mg/L	465 ± 15.2	375	338
Al	mg/L	367 ± 21.2	120	0.53
Mn	mg/L	85.4 ± 3.8	23	13
Na	mg/L	33.1 ± 0.92	19	5005
Si	mg/L	28.1 ± 1.02	10	4
Zn	mg/L	9.8 ± 0.01	1.3	0
B	mg/L	-	-	2194
K	mg/L	-	242	-
**Anions**				
SO^2−^_4_	mg/L	11,500 ± 29.0	7820	10,400

In the case of AMD treated with green tea extract, 62% of the target element Fe was removed as nano-iron. When sodium borohydride was used as a reductant, 99.9% of Fe was removed as nano-iron. This is because sodium borohydride has a higher FRAP value—about five times higher than green tea extract. This means that sodium borohydride is a better reducing agent for iron and most of the other elements in the AMD solution. Table 4 also revealed that some of the major elements found in AMD co-precipitated with Fe during its removal from AMD. This study aligns with previous studies that focused on the removal of Fe (in other forms) from AMD (Wang et al. 2019a, b). Even though sodium borohydride was effective in removing Fe from AMD (nZVI), the reductant significantly increased the concentrations of boron (B = 2194 mg/L) and sodium (Na = 5005 mg/L) in the AMD solution, hence contaminating the solution further. In line with this investigation, the study could open room for further exploration of using possible cheap plant extracts with strong antioxidant capacity to reduce more Fe in AMD.

### Catalytic testing of *iron* nanoparticles extracted from AMD in the treatment of methylene blue solution

This study utilized iron nanoparticles synthesized from AMD to decolorize a simulated methylene blue solution. This was to determine if the AMD-synthesized iron nanoparticles (GTNI 0.5 or SBNI 0.6) were active as catalysts. GTNI 0.5 iron nanoparticles (Fe-NPs) were synthesized from AMD using green tea extract, whereas SBNI 0.6 Fe-NPs were synthesized from AMD using sodium borohydride (a chemical reductant). In the treatment of the methylene blue solution, the pH, mass of the catalyst, and contact time were considered for optimization. During this process, the synthesized iron nanoparticles in the presence of H_2_O_2_ generate free radicals that disrupt the chromophore link of methylene blue, resulting in a decolorized solution.

#### Effect of pH

**Fig. 6:**
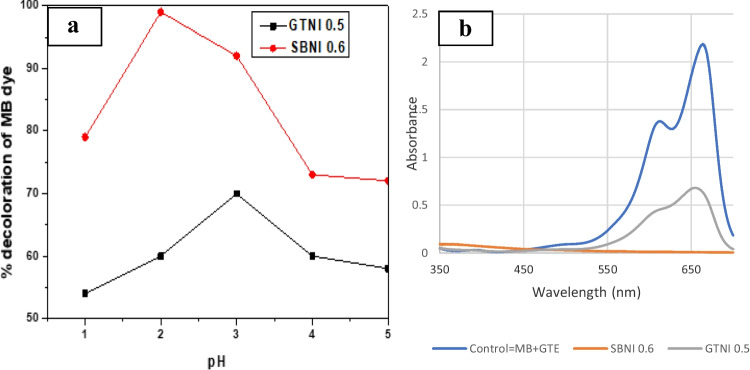
**a** Bar chart; **b** UV spectra, showing the effect of pH on the percentage decoloration of methylene blue. MB + GTE (methylene blue + green tea extract)

Figure 6 shows the effect of pH in the range of 1–5 on decolorizing 100 mL of methylene blue in solution using 5 mg of GTNI 0.5 or SBNI 0.6 for 180 min in a shaker.

At a pH of 2 or 3, the optimum decoloration of MB in solution using SBNI 0.6 or GTNI 0.5 was 99% or 71%, respectively. The reactivity of GTNI 0.5 decreased as the pH increased from 3 to 5, whereas the activity of SBNI 0.6 decreased as the pH increased from 2 to 5. The results showed that the iron nanoparticles synthesized from AMD were active as Fenton catalysts for the decoloration of methylene dye in an acidic environment. Badmus et al. (2018) reported using an acidic medium during the degradation of orange (II) sodium salt in the solution. The UV spectra confirmed the graph in Fig. 6a and demonstrated that GTNI 0.5 catalyst had optimum performance for MB decoloration at pH = 3, whereas SBNI 0.6 had optimum performance at pH = 2. The optimum pH values were then applied during the optimization of the dosage of catalyst and time in the Fenton reaction.

#### Effect of the dosage of the catalyst

**Fig. 7:**
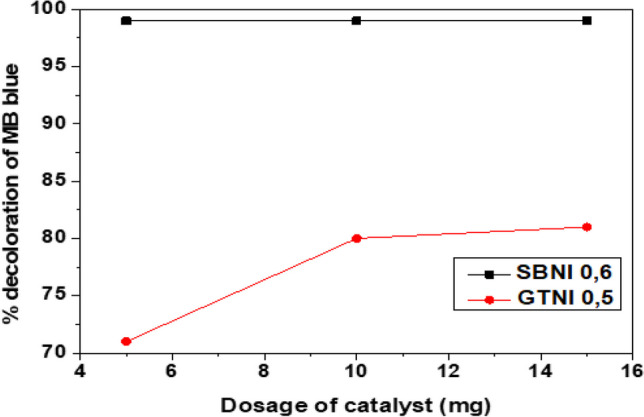
Effect of catalyst dose on the percentage decoloration of MB in solution

The effect of varying the catalyst dose (GTNI 0.5 or SBNI 0.6) on the percentage decoloration of methylene blue in the solution at optimum pH (of 2 or 3) was investigated. Figure 7 depicts the result.

Figure 7 showed that 5 mg of SBNI 0.6 catalyst was established to achieve 99% decoloration of MB in the solution. Adding higher dosages of SBNI 0.6 (10 or 15 mg) did not increase or decrease the outcome. In the case of using the GTNI 0.5 catalyst, a higher dosage of 10 mg was applied to achieve 81% decoloration of MB. The highest dosage (15 mg) of GTNI 0.5 had an insignificant change in the decoloration performance. It is worth noting that SBNI 0.6 decolorizes MB in the solution better than GTNI 0.5, even at a lower dosage. This is because the SBNI 0.6 iron nanoparticles have no polyphenolic coating, which could alter their performance, whereas the polyphenolic coating on the GTNI 0.5 nanoparticles reduced its performance. This is because the polyphenol compound and the iron nanoparticles have a strong intermolecular bond between them (Ravikumar et al. 2016).

#### Effect of the contact time

**Fig. 8:**
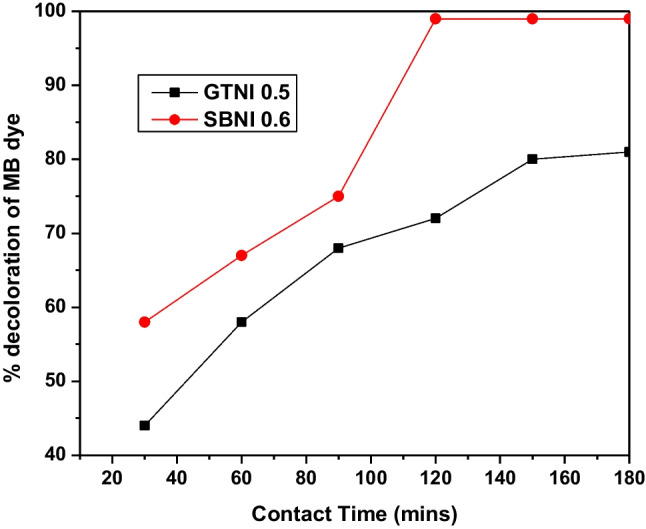
Effect of the contact time on the percentage decoloration of methylene blue using GTNI 0.5 (10 mg) or SBNI 0.6 (5 mg) iron nanoparticles

By varying the contact time in an interval of 30 min at optimum pH (2 or 3) and dosage of catalyst, GTNI 0.5 or SBNI 0.6 were used to investigate the effect of contact time on the percentage decoloration of MB blue in solution. Figure 8 depicts the information.

Figure 8 showed that the extent of decoloration of methylene blue by GTNI 0.5 or SBNI 0.6 was noticed to increase to a maximum value as contact time increased. The decoloration of MB becomes constant with an increase in contact time of 120 or 150 min for SBNI 0.6 or GTNI 0.5, respectively. Based on this data, the equilibrium time for the decoloration experiments was 120 or 150 min. The contact time optimization of these experiments contradicted the use of 3 h as an ideal time for MB decoloration.

## Conclusion

This study demonstrated that an environmentally friendly approach (using green tea extract) and a chemical approach (using sodium borohydride) can be used to synthesize high-quality iron nanoparticles from AMD. The chemically synthesized nanoparticles were unstable under air, and their accompanied supernatant was toxic, containing a high concentration of Na and B. The green tea synthesized nanoparticles were stable under air due to polyphenol coating, and their accompanied supernatant was less toxic, making it a preferred reductant for nanoiron synthesis. Both types of iron nanoparticles were active as Fenton-like catalysts for the decoloration of methylene blue (MB) in solution. However, the chemically synthesized nanoparticles performed about 20% better than the green tea synthesized nanoparticles. In line with this investigation, this study lays the groundwork for looking into cheaper, locally sourced renewable materials that could be used to synthesize iron nanoparticles from Fe-rich waste streams like AMD. This could make the treatment of AMD more cost-effective and long-lasting.

## Data Availability

This article includes all other relevant data generated and analysed during this study. We will make the XRD data available upon request.
